# Polygenic association with severity and long-term outcome in eating disorder cases

**DOI:** 10.1038/s41398-022-01831-2

**Published:** 2022-02-16

**Authors:** Therese Johansson, Andreas Birgegård, Ruyue Zhang, Sarah E. Bergen, Mikael Landén, Liselotte V. Petersen, Cynthia M. Bulik, Christopher Hübel

**Affiliations:** 1 Department of Medical Epidemiology and Biostatistics, Karolinska Institutet, Stockholm, Sweden; 2 Department of Immunology, Genetics and Pathology, Science for Life Laboratory, Uppsala University, Uppsala, Sweden; 3 Centre for Women’s Mental Health during the Reproductive Lifespan – Womher, Uppsala University, Uppsala, Sweden; 4 Institute of Neuroscience and Physiology, University of Gothenburg, Gothenburg, Sweden; 5 National Centre for Register-based Research, Department of Economics and Business Economics, Aarhus University, Aarhus, Denmark; 6 Department of Psychiatry, University of North Carolina at Chapel Hill, Chapel Hill, NC USA; 7 Department of Nutrition, University of North Carolina at Chapel Hill, Chapel Hill, NC USA; 8 Social, Genetic & Developmental Psychiatry Centre, Institute of Psychiatry, Psychology & Neuroscience, King’s College London, London, UK; 9 UK National Institute for Health Research (NIHR) Biomedical Research Centre for Mental Health, South London and Maudsley Hospital, London, UK

**Keywords:** Prognostic markers, Psychiatric disorders, Personalized medicine

## Abstract

About 20% of individuals with anorexia nervosa (AN) remain chronically ill. Therefore, early identification of poor outcome could improve care. Genetic research has identified regions of the genome associated with AN. Patients with anorexia nervosa were identified via the Swedish eating disorder quality registers Stepwise and Riksät and invited to participate in the Anorexia Nervosa Genetics Initiative. First, we associated genetic information longitudinally with eating disorder severity indexed by scores on the Clinical Impairment Assessment (CIA) in 2843 patients with lifetime AN with or without diagnostic migration to other forms of eating disorders followed for up to 16 years (mean = 5.3 years). Second, we indexed the development of a severe and enduring eating disorder (SEED) by a high CIA score plus a follow-up time ≥5 years. We associated individual polygenic scores (PGSs) indexing polygenic liability for AN, schizophrenia, and body mass index (BMI) with severity and SEED. After multiple testing correction, only the BMI PGS when calculated with traditional clumping and *p* value thresholding was robustly associated with disorder severity (*β*_PGS_ = 1.30; 95% CI: 0.72, 1.88; *p* = 1.2 × 10^–5^) across all *p* value thresholds at which we generated the PGS. However, using the alternative PGS calculation method PRS-CS yielded inconsistent results for all PGS. The positive association stands in contrast to the negative genetic correlation between BMI and AN. Larger discovery GWASs to calculate PGS will increase power, and it is essential to increase sample sizes of the AN GWASs to generate clinically meaningful PGS as adjunct risk prediction variables. Nevertheless, this study provides the first evidence of potential clinical utility of PGSs for eating disorders.

## Introduction

Eating disorders are complex psychiatric conditions that arise from a combination of genetic and environmental factors [1]. Anorexia nervosa (AN) is among the most serious and deadly of all psychiatric disorders [2] as only 30% of patients achieve remission [3] and 20% remain chronically ill [4]. A severe and enduring eating disorder (SEED) describes those with AN or bulimia nervosa (BN) with chronic symptoms, treatment non-response [5–7], and long duration of illness, variably defined as five [8], six [9], seven [10, 11], or ten [12] years.

In AN, concurrent anxiety or depressive symptoms, psychosocial difficulties [13], long duration of illness prior to hospitalisation, low BMI, and inadequate weight gain during hospitalisation are associated with poor outcome in general and 21 years after initial hospitalisation [14, 15]. In BN, findings are mixed with a high frequency of compensatory behaviours [16] and comorbid psychiatric diagnoses associated with poor outcome [17–19], whereas perfectionism, obsessionality, anxiety, and genetic factors may increase the likelihood of developing a SEED [20]. Studies to identify predictors of poor outcome for binge-eating disorder (BED) have not had adequate statistical power [21, 22].

The largest genome-wide association study (GWAS) published as of 2020 associated eight risk loci with AN and indicated a genetic sharing between AN and BMI (*r*_g_ = −0.32) and between AN and schizophrenia (SCZ; *r*_g_ = 0.25), corroborating observed comorbidity and familiality [23] in clinical and epidemiological studies [24–26]. These findings implicate both anthropometric and psychiatric factors in the origin of AN [27]. Individual genetic liability to, for example, high BMI can be expressed as polygenic scores (PGSs). PGSs are calculated for each individual by weighting the genomic variants the individual carries across the whole genome by effect sizes obtained from GWASs. PGSs thereby capture the polygenic signal of a given trait [28]. Individuals with high PGSs carry more risk variants and are hypothesised to be more likely to develop a trait or disorder. Robust findings from GWAS are used to construct PGS for psychiatric disorders [29–31] and evaluate shared genetic risk between phenotypes [32–34].

In patients followed up on average 5.3 years in quality registers covering specialised eating disorder care across Sweden [35], we defined two different outcomes: first, at the timepoint when the patients additionally joined the Anorexia Nervosa Genetics Initative (ANGI), we defined severity as the total score on the 16-item Clinical Impairment Assessment (CIA) questionnaire. The CIA measures secondary psychosocial impairment due to eating disorder symptoms. Second, we created an index of SEED marked by a high CIA score plus a follow-up time ≥5 years. Based on the literature, we selected three PGSs and explored their association with severity or a SEED: PGS for AN, SCZ, and BMI. First, we hypothesised that an individual’s eating disorder severity and risk for a SEED would be influenced by a high AN PGS (i.e., higher genetic loading associated with greater severity). Second, based on the reported high genetic correlations between AN and SCZ [27], the seven times increased risk of SCZ and the familial liability of SCZ, in patients with eating disorders compared with the general population [23], we hypothesised that greater genetic liability to SCZ separately would be associated with greater severity and SEED. Furthermore, we chose the SCZ GWAS as it currently represents the best statistically powered GWAS of any psychiatric disorder genetically correlated with AN, shows high heritability, and may index genetic liability to general psychopathology. Third, given the observed negative genetic correlation between AN and BMI [27], and the association between low phenotypic BMI and poor outcome [36–38], we hypothesised that a BMI PGS would separately be associated with severity and the development of a SEED. Furthermore, we chose the BMI PGS because it is currently the largest GWAS available of anthropometric traits genetically associated with AN, and, therefore, offers the greatest statistical power. Results could inform whether PGSs might serve as a useful adjunct tool in predicting severity or a SEED.

## Methods

### Sample

Our study includes Swedish AN cases from ANGI [39] whose genetic analytic methods have been described [27]. Participants were recruited for genotyping into ANGI via Swedish treatment centres and two national registers: (1) Riksät, the national quality register established in 1999, including patients treated for AN, BN, or eating disorders not otherwise specified (EDNOS) [40] and (2) Stepwise, the internet-based clinical quality assurance database for specialised eating disorder care, established in 2005. Stepwise includes all Riksät and additional variables and was used by an expanding number of treatment centres until 2015. Patients are registered into Riksät/Stepwise if (1) they are medically/self-referred to a Swedish treatment centre, (2) treatment is intended, and (3) an eating disorder diagnosis has been established (Table 1). Patients were first entered into either register 1999–2016 and recontacted for participation in ANGI 2013–2016. Therefore, for some participants, the follow-up time between entering the treatment register and being recruited into ANGI was less than a month (Table 2). We calculated follow-up time as difference in years between the year at first registration and the year when participants were recontacted for recruitment into ANGI, covering a follow-up period of 0–16 years. Our analyses comprised a total of 2843 individuals aged 10–66 years with 45 males (1.6% of the total sample; Table 2).

**Table 1:** Lifetime eating disorder diagnoses registered in Swedish quality registers Riksät/Stepwise among individuals with a lifetime diagnosis of anorexia nervosa (AN) included in our target sample (*n* = 2834).

Eating disorder diagnoses registered in Stepwise/Riksät		
	*n* (cases)	% of total
AN restricting only	976	34.3
AN binge-eating/purging only	260	9.2
Broad AN only (without amenorrhoea and weight criterion)	603	21.2
AN + bulimia nervosa	522	18.4
AN + binge-eating disorder	45	1.6
AN + EDNOS	95	3.3
AN + purging disorder or EDNOS 5*	342	12.0
**Total**	2843	

*AN* anorexia nervosa, *BN* bulimia nervosa, *BED* binge-eating disorder, *EDNOS* eating disorder not otherwise specified, *PD* purging disorder.

**Table 2:** Descriptive statistics of quantitative demographic and clinical characteristics of the target sample (*n* = 2843) of specialised eating disorder care in Sweden.

	Mean	SD	Median	Min	Max	Total missing
*Self-reported age at first eating disorder symptom*						
AN restricting only	15.93	4.38	15	6	58	
AN binge-eating/purging only	15.64	4.13	15	6	44	
Broad AN only (without amenorrhoea and weight criterion)	15.31	3.78	15	6	45	
AN + bulimia nervosa	15.55	3.77	15	5	33	
AN + binge-eating disorder	17.31	6.74	16	10	55	
AN + EDNOS	15.68	4.30	15	6	36	
AN + purging disorder or EDNOS 5^b^	15.60	3.93	15	6	36	
						155
*Age at first registration*						
AN restricting only	19.44	6.68	18	10	61	
AN binge-eating/purging only	21.79	7.00	20	13	60	
Broad AN only (without amenorrhoea and weight criterion)	20.50	7.35	18	11	66	
AN + bulimia nervosa	25.33	7.98	23	13	66	
AN + binge-eating disorder	26.27	9.44	24	16	61	
AN + EDNOS	22.20	8.53	20	12	60	
AN + purging disorder or EDNOS 5^a^	23.21	8.62	21	11	60	
						0
*Body mass index (kg/m*^*2*^*)*						
AN restricting only	15.91	1.39	16	12	22	
AN binge-eating/purging only	16.52	1.49	17	13	25	
Broad AN only (without amenorrhoea and weight criterion)	18.63	1.89	18	14	31	
AN + bulimia nervosa	21.67	3.78	21	14	44	
AN + binge-eating disorder	24.38	6.35	22	16	44	
AN + EDNOS	19.63	4.40	19	13	40	
AN + purging disorder or EDNOS 5^a^	19.89	2.38	20	14	34	
						15
*Clinical Impairment Assessment (CIA) global score*						
AN restricting only	17.13	12.94	14	0	48	
AN binge-eating/purging only	21.17	14.19	21	0	48	
Broad AN only (without amenorrhoea and weight criterion)	18.58	12.73	17	0	48	
AN + bulimia nervosa	19.84	12.83	19	0	48	
AN + binge-eating disorder	19.33	13.84	21	0	46	
AN + EDNOS	14.51	11.30	11	0	40	
AN + purging disorder or EDNOS 5^a^	19.88	13.17	20	0	48	
						0
*Follow-up time*^b^						
AN restricting only	5.31	3.34	5	0	15	
AN binge-eating/purging only	5.38	3.64	5	0	14	
Broad AN only (without amenorrhoea and weight criterion)	4.59	3.25	4	0	16	
AN + bulimia nervosa	5.19	3.69	4	0	16	
AN + binge-eating disorder	5.58	3.55	5	0	14	
AN + EDNOS	4.34	2.82	3	0	12	
AN + purging disorder or EDNOS 5^a^	3.91	3.14	3	0	14	
						0

^a^Anorexia nervosa.

^b^EDNOS 5 = Eating disorder not otherwise specified category 5, includes those patients that repeatedly chew and spit food, without swallowing, large amounts of food.

^c^Follow-up time was calculated as difference in years between year at first registration and the year when participants were recontacted for recruitment into the Anorexia Nervosa Genetics Initiative (ANGI).

### Clinical diagnosis

To be classified as a case in ANGI, a lifetime DSM-IV AN diagnosis (amenorrhoea was not required) was confirmed based on answers to the ED100K-v1 questionnaire [39] or by a clinical AN diagnosis registered in the national registers. Importantly, all individuals in our study had a lifetime AN diagnosis, but may also have been diagnosed with an additional eating disorder. This means we could delineate a persistent AN group from a AN with mixed eating disorder presentation group, representing the diagnostic crossover common in eating disorders [41] (Table 1). In Riksät, diagnoses are based on clinician interviews and clinical observation until 2013 when the DSM-IV-based Structured Eating Disorder Interview (SEDI) [42] was also implemented. In Stepwise, the Structured Clinical Interview for DSM-IV Axis I disorders (SCID-I), eating disorder diagnostic research version (module H) [43] for adults ≥18 years was administered from 2005 until August 2008 alongside the MINIkid version 2.1 for DSM-IV (i.e., Modules S and T) [44] for individuals <18 years. The DSM-IV-based SEDI [42] has been used since August 2008.

### Treatment centres

We grouped the 41 treatment centres into five categories reflecting their geographic locations across Sweden. We excluded Stockholm from “Svealand” to create a more equal distribution of participants across categories, reflecting differences between rural and urban regions. Most patients were first registered in the region of Stockholm (32.3%), followed by Västra Götaland (22.3%), Östergötland (22.0%), and Svealand (16.0%), while the fewest patients were registered in Norrland (7.4%). About 29% of patients at treatment centres in Stockholm received an AN restricting diagnosis.

### Ethics

All participants provided written informed consent for participation in ANGI. The Swedish component of ANGI was approved by the regional Ethical Review Board in Stockholm (dnr: 2013/112-31/2) and the amendments (2014/1563 and 2016/1852-32).

### Eating disorder severity as measured by the clinical impairment assessment

The 16-item questionnaire measures the severity of secondary psychosocial impairment due to eating disorder features during the previous 28 days [35], covering three domains: emotional, social, and cognitive functioning. Each item is rated on a 4-point Likert scale with 0 = ‘not at all’, 1 = ‘a little’, 2 = ‘quite a bit’, and 3 = ‘a lot’. Summing all items yields a total severity score ranging from 0 to 48, with higher scores indicating greater impairment (Supplementary Fig. S1A–C). More details on the questionnaire are in the Supplementary Methods. Correlations between items were assessed using polychoric correlations and polyserial correlations between the sum scores (i.e., global and domain-specific scores) and the 16 items [45]. Pearson’s correlations were used to estimate the association between the sum scores (Supplementary Fig. S2).

### SEED definition

With the assumption that a CIA total score ≥18 is associated with case status [46], we defined our binary dependent SEED variable as CIA score ≥18 and a follow-up time ≥5 years (i.e., years between initial registration and ANGI recruitment) independent of eating disorder diagnosis or diagnostic crossover. We compared patients with a SEED with those having a CIA score <18 and follow-up time ≥5 years in logistic regressions. As treatment resistance is part of the proposed definition of a SEED [7] it is important to note that all patients registered in Risksät/Stepwise have undergone at least one treatment attempt, qualifying them for a SEED.

### PGS calculation: clumping and thresholding

The PGSs for AN (after excluding Swedish participants; *n* = 4118) [39] and SCZ [31] were based on genome-wide association data from the Psychiatric Genomics Consortium (PGC), and a PGS for BMI based on data from the Genetic Investigation of ANthropometric Traits (GIANT) consortium and the UK Biobank [47]. SNPs that were present exclusively in either the GWAS summary statistics or the ANGI dataset as well as ambiguous SNPs were removed before PGS calculation using PRSice, version 2.2.3 [48]. Linkage disequilibrium (LD) among SNPs was accounted for by clumping. The genetically independent SNP with the smallest *p* value in each 250 kilobase window of all those in LD was retained as the index SNP (*r*^2^ > 0.1). We calculated PGSs at different *p* value thresholds (*p* values: 5 × 10^−8^, 1 × 10^−5^, 0.001, 0.01, 0.05, 0.1, 0.2, 0.3, 0.4, 0.5, 1) for sensitivity analyses. The main results are based on the PGSs at a *p* value threshold of 0.001 as this PGS explained most of the variance of the CIA score in the model including the AN PGS (*R*^2^ = 0.175%). We also chose to report this threshold in the models including the BMI or SCZ PGSs to facilitate comparison across models. As sensitivity analysis, we present results for all other *p* value thresholds. We included standardised PGS as continuous variables, reporting results as one unit change per one standard deviation higher PGS.

### Polygenic risk score continuous shrinkage (PRS-CS)

We observed inconsistent results for the AN PGS across the different *p* value thresholds at which they were generated. Therefore, we used PRS-CS as a sensitivity analysis to further investigate the robustness of our results. PRS-CS does not rely on *p* value thresholding and is a Bayesian approach to calculate PGS. It applies two shrinkage parameters to the original effect sizes of the discovery GWAS: a global scaling parameter shared across all effect sizes and a local, marker-specific parameter, resulting in a global-local scale mixtures of normals. In an extreme case, where the local shrinkage would be one, the model would be a Ridge regression and all effect sizes were shrunk by the global parameter only. However, the local shrinkage parameter allows heterogeneity in the scales of the effect sizes and the global parameter controls the sparsity of the model. In other words, small effects are shrunk towards zero, but large effect sizes are less affected. In summary, the PRS-CS method calculates a reweighted PGS assuming a different degree of involvement of different SNPs across the genome. No selection of a *p* value threshold is needed. The reweighted PGS was used as an explanatory variable in our regression models. Correlations between the thresholding-based and PRS-CS-based PGS are presented in Supplementary Fig. S3A–C.

### Statistical analysis

We associated the three PGSs for AN, SCZ, and BMI with disorder severity measured by the CIA total score as a continuous dependent variable (i.e., linear regression) and with SEED (i.e., logistic regression), resulting in six regression models. To avoid collinearity among independent variables, we excluded highly correlated variables (i.e., *r* > 0.80; Supplementary Fig. S4). In our main model (Table 3), we fitted the PGS as the independent variable and adjusted for potential population stratification by including the first 10 ancestry-informative principal components (PCs), and controlled for nesting within our sample by including treatment region [49]. Using PLINK 2.0, we calculated PCs based on pruned genotype data [50]. Additionally, we included age, sex, and follow-up time. For all model details including justification, see Table 3. Mendelian randomisation studies imply a bidirectional causal relationship between AN and low BMI; hence, adjusting for BMI in models including patients with AN may introduce a collider [27]. Therefore, we investigated BMI in an additional model (logistic model 4 and linear model 5). We investigated three different PGSs and two outcomes and therefore adjusted our *α* threshold using the Bonferroni method $\alpha = \frac{{0.05}}{6} = 0.008$ to account for the multiple tests performed.

**Table 3:** Regression models fitted.

Linear regression	Logistic regression	Variables	Justification
Model 1	Model 1	CIA or SEED ~ PGS + PCs 1–10 + treatment region	Population stratification and nesting
Model 2	Model 2	Model 1 + Age	Older age at treatment initiation is associated with poor outcome [49]
Model 3	**Model 3** (main model)	Model 2 + Sex + AN presentation^a^	Sex differences in presentation [50] and prevalence [51] of eating disorders
**Model 4** (main model)	–	Model 3 + Follow-up time^b^	To evaluate disorder severity independent of follow-up time (years between first registration and ANGI recruitment).
Model 5	Model 4	Model 3 + Follow-up time^b^ + BMI	Low body weight as predictor of poor outcome [14, 15, 22]
Model 6	Model 5	Model 3 + Follow-up time^b^ + Self-reported age at first eating disorder symptom	Early age at onset is indicative of a better outcome [22] and hormonal changes are associated with disorder onset [52]

*ANGI* Anorexia Nervosa Genetics Initiative, *CIA* Clinical Impairment Assessment [35], *BMI* body mass index, *PC* principal component, *PGS* polygenic score, *SEED* severe and enduring eating disorder.

^a^Only adjusted for eating disorder diagnosis when including all participants, irrespective of clinically ascertained eating disorder diagnosis additional to AN.

^b^Only adjusted for when analysing disorder severity as a continuous variable. Defined as years between first treatment registration in the quality registers and recruitment into ANGI.

Model 3 is the main model in the logistic regression analysis and model 4 is the main model in the linear regression. The other models are sensitivity analyses.

### Exclusion

From analysis including BMI as a covariate, 15 patients were excluded due to missing data, and 155 patients were excluded because of missing retrospectively self-reported age at first eating disorder symptom. From the logistic models, we excluded follow-up time as a covariate since follow-up time is part of the SEED outcome definition (i.e., longer than 5 years). This means that the logisitic regression only includes patients with a follow-up ≥ 5 years. Therefore, the total sample size for the logistic regression analysis was *n* = 1334.

### Sensitivity analyses

Approximately 35% of the individuals in our target sample (Table 1) were not diagnosed with AN when they were first registered into Riksät/Stepwise. Because this mixed presentation of an eating disorder in one individual may influence the association between PGS and outcome, we used sensitivity analyses to evaluate the effect of diagnostic heterogeneity in the eating disorder presentation. We created four different samples: first, the whole sample including individuals with AN and a mixed presentation of other eating disorder diagnoses, second, individuals with AN, including a clinical AN restricting, binge-eating/purging subtype, or AN without amenorrhoea and weight criterion, third, only individuals with a clinical AN restricting diagnosis, as this may represent a unique phenotype, fourth, only individuals with AN (both subtypes) with a low BMI (≤17.5 kg/m^2^). The subsamples including all AN subtypes or only the AN restricting subtype did not require adjustment for the eating disorder diagnosis. In this case, we fitted five logistic regression models.

## Results

Participants with AN restricting subtype had the lowest average BMI (15.9 kg/m^2^), were the youngest (age = 19.4 years) at first registration, and had the lowest CIA total score (mean CIA = 17; i.e., least severe). Self-reported age at first eating disorder symptom was similar across all groups except for individuals with lifetime co-occurring BED, who reported the oldest age at experiencing their first eating disorder symptom (age = 17.3 years). Follow-up time was on average the same across all groups (follow-up time ≈ 5 years) except for participants with AN plus purging disorder (follow-up time = 3.91 years; Table 2). Supplementary Fig. 1A–C summarise responses to each CIA item in all three samples.

### Association of PGSs with eating disorder severity as measured by the CIA

#### AN PGS and eating disorder severity

**Fig. 1:**
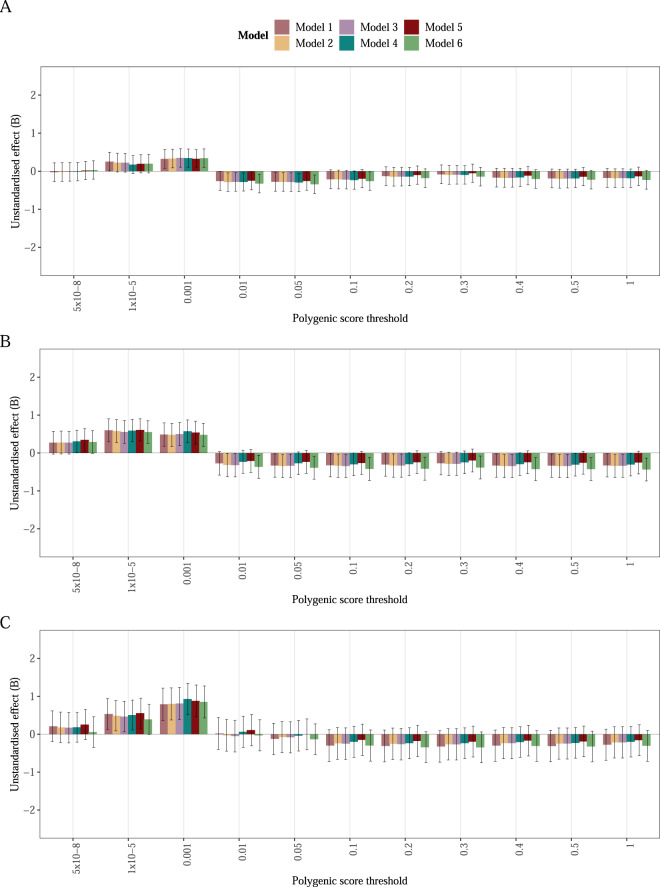
Anorexia nervosa polygenic score (PGS) associated with disorder severity measured as Clinical Impairment Assessment (CIA) total score. Bars represent beta estimates ± standard errors. **A** is based on 2843 individuals with anorexia nervosa and additional eating disorder diagnoses during their lifetime. **B** is based on 1839 individuals with any clinically ascertained anorexia nervosa diagnosis. **C** is based on 976 individuals with a clinically ascertained anorexia nervosa restricting subtype. The estimates in model 4 are adjusted for covariates included in the main model: Principal components 1–10, treatment region, age, sex, follow-up time and eating disorder diagnosis when including individuals with any eating disorder diagnosis (*n* = 2843).

After multiple testing correction (*α* = 0.008), the AN PGS was not associated with disorder severity (Supplementary Table S1) measured as the CIA total score in either the full sample with clinically ascertained AN cases with a mixed presentation (*n* = 2843, Fig. 1A), or the AN cases subsample (i.e., AN restricting, AN binge-eating/purging, Broad AN; *n* = 1839, Fig. 1B), or the subsample of individuals with the AN restricting subtype (*n* = 976, Fig. 1C), or the subsample of individuals with low weight AN. Adjustment of our models did not change the association between the AN PGS and the CIA total score. Moreover, after shrinking the effect sizes and re-weighting the PGS with PRS-CS, the association remained non-significant (Supplementary Table S2).

#### SCZ PGS and eating disorder severity

**Fig. 2:**
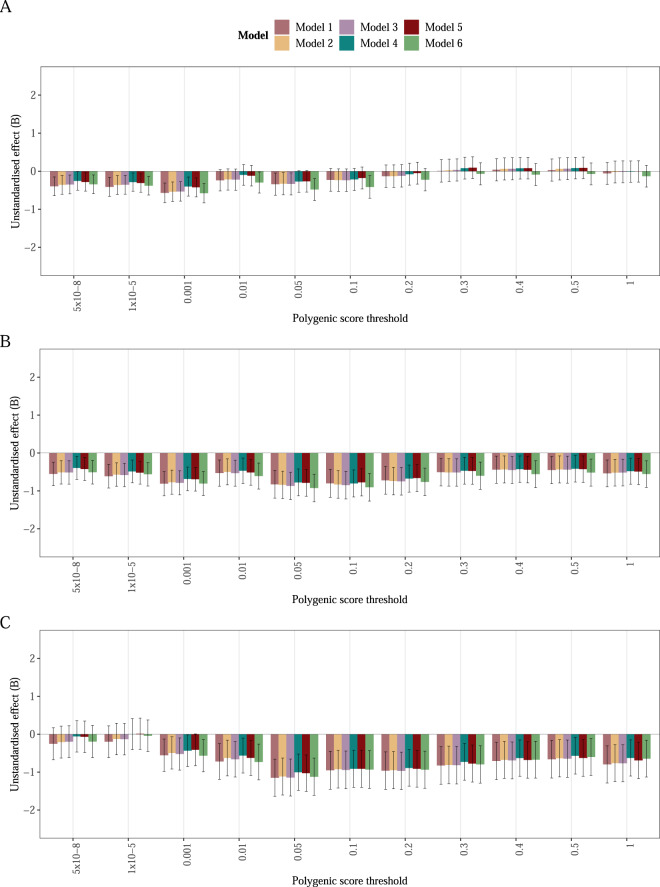
Schizophrenia polygenic score (PGS) associated with disorder severity measured as Clinical Impairment Assessment (CIA) total score. Bars represent beta estimates ± standard errors. **A** is based on 2843 individuals with anorexia nervosa and additional eating disorder diagnoses during their lifetime. **B** is based on 1839 individuals with any clinically ascertained anorexia nervosa diagnosis. **C** is based on 976 individuals with a clinically ascertained anorexia nervosa restricting subtype. The estimates in model 4 are adjusted for covariates included in the main model: Principal components 1–10, treatment region, age, sex, follow-up time and eating disorder diagnosis when including individuals with any eating disorder diagnosis (*n* = 2843).

The same result was observed for the SCZ PGS: no association remained significant at the *α* = 0.008 (Fig. 2A–C and Supplementary Table S3). We did not observe any significant association by subsample, model adjustment, or PRS calculation method.

#### BMI PGS and eating disorder severity

**Fig. 3:**
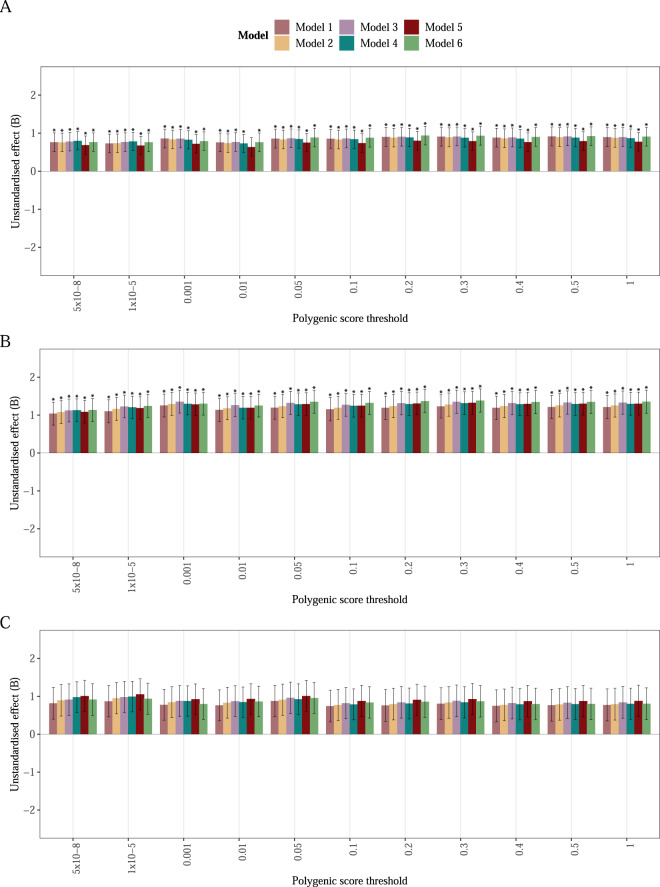
Body mass index (BMI) polygenic score (PGS) associated with disorder severity measured as Clinical Impairment Assessment (CIA) total score. Bars represent beta estimates ± standard errors. **A** is based on 2843 individuals with anorexia nervosa and additional eating disorder diagnoses during their lifetime. **B** is based on 1839 individuals with any clinically ascertained anorexia nervosa diagnosis. **C** is based on 976 individuals with a clinically ascertained anorexia nervosa restricting subtype. The estimates in model 4 are adjusted for covariates included in the main model: Principal components 1–10, treatment region, age, sex, follow-up time and eating disorder diagnosis when including individuals with any eating disorder diagnosis (*n* = 2843). The asterisk (*) denotes statistically significant results (*α* = 0.008).

The BMI PGS showed a positive association with eating disorder severity as measured by the CIA (Fig. 3 and Supplementary Table S4). In the full sample including clinical AN cases with a mixed presentation, one standard deviation greater BMI PGS was associated with a 0.83 (95% CI: 0.36, 1.30; *p* = 5.5 × 10^−4^) greater CIA total score (Fig. 3A). Limiting the sample to AN only cases strengthend the association: one standard deviation greater BMI PGS was associated with 1.30 (95% CI: 0.72, 1.88; *p* = 1.2 × 10^−5^) greater CIA total score (Fig. 3B). In the AN restricting subtype subsample or the low weight AN subsample, the association did not remain significant at our α threshold (Fig. 3C). The results in the full sample and the AN only sample were consistent across all *p* value thresholds at which the PGS was generated when using clumping and thresholding. Additionally, the results remained largely the same with varying adjustment of our models. However, after shrinking the effect sizes and re-weighting the PGS with PRS-CS, the association did not remain significant (Supplementary Table S2).

### Association of PGS with risk of SEED

We tested if PGSs for any of the three selected traits were associated with SEED, defined as those individuals with a CIA score ≥18 and a follow-up time ≥5 years.

#### AN PGS and SEED

**Fig. 4:**
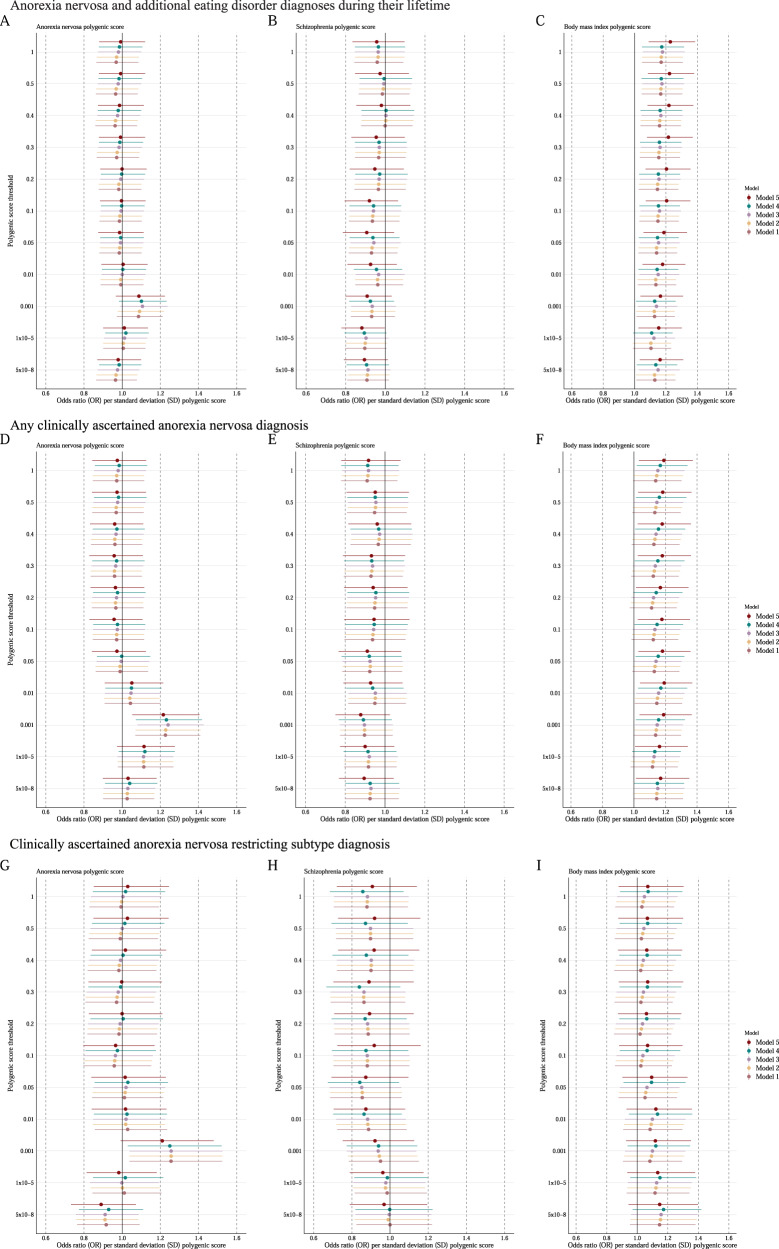
Polygenic scores (PGSs) associated with a severe and enduring eating disorder (SEED). **A–C** 1334 individuals with anorexia nervosa and additional eating disorder diagnoses during their lifetime (*n* SEED = 528, *n* non-SEED = 806). Figure 4A shows the association with the anorexia nervosa PGS, Fig. 4B with the schizophrenia PGS, and Fig. 4C with the BMI PGS. **D**–**F** 901 individuals with any clinical anorexia nervosa diagnosis (*n* SEED = 337, *n* non-SEED = 564). **D** shows the association with the anorexia nervosa PGS, **E** with the schizophrenia PGS, and **F** with the BMI PGS. **G**–**I** 509 individuals with a clinical anorexia nervosa restricting subtype diagnosis (*n* SEED = 178, *n* non-SEED = 331). **G** shows the association with the anorexia nervosa PGS, **H** with schizophrenia PGS, and **I** with the BMI PGS. The estimates in model 3 are adjusted for covariates included in the main model: Principal components 1–10, treatment region, age, sex and eating disorder diagnosis when including individuals with any eating disorder diagnosis. Dots represent odds ratios (ORs) and error bars 95% confidence intervals.

In the full sample with a mixed AN presentation, the AN PGS was not significantly associated with a SEED (*n* = 1334 Fig. 4A). When we restricted the analysis to individuals with AN only (i.e., AN restricting, AN binge-eating/purging, Broad AN; *n* = 901), a one standard deviation greater AN PGS was associated with 24% higher odds of being classified as a SEED (95% CI: 1.08, 1.43; *p* = 0.002; Fig. 4D and Supplementary Table S5). The association did not remain significant in the AN restricting subsample (*n* = 509; Fig. 4G) or the low weight subsample. We detected the association only at a PGS generation *p* value threshold of 0.001; however, the association was independent of adjustment by other variables. The association was not detected when calculating the PGS with PRS-CS (Supplementary Table S6).

#### SCZ PGS and SEED

We found no statistically significant associations between SCZ PGSs and SEED in either the full or our subsamples of diagnostic subgroups (Fig. 4B, E, H and Supplementary Table S7). Model adjustement or a different PGS calculation method did not change the result.

#### BMI PGS and SEED

We found no statistically significant associations between BMI PGSs and SEED in either the full or our subsamples of diagnostic subgroups at our main PGS generation *p* value threshold of 0.001. However, when including self-reported age at first eating disorder symptom (model 5) as an additional explanatory variable and when including more SNPs in the PGS (i.e., generation *p* value thresholds from 0.05 until 1), the BMI PGS was significantly associated with SEED only in the full sample with a mixed AN presentation with ORs ranging from 1.19 to 1.23; Fig. 4C and Supplementary Table S8). Overall, these results were inconsistent.

## Discussion

Eating disorders are often chronic [4] and no biomarkers exist that identify individuals at risk for an eating disorder, let alone for a protracted illness course. Our results suggest that a PGS indicating genetic liability to BMI may be a useful adjunct tool in predicting who is at risk for developing a severe an enduring form of the illness. Additionally, if the sample size for the AN GWAS increases, and the AN PGS may become more statistically powerful, the AN PGS should be re-assessed for outcome prediction.

Several recent reviews have highlighted the potential clinical application of PGSs in psychiatry, as well as cautions about their overinterpretation [51, 52]. Research on other psychiatric disorders such as depression has shown that the cumulative influence of multiple genetic variants is associated with chronic depressive symptoms [53]. Similarly, a high SCZ PGS is associated with poor treatment response, suggesting that polygenic burden may impact treatment resistance [54].

Our results represent the first indication that an AN PGS may have clinical utility in the future. PGSs have become increasingly robust as the sample sizes of their source GWASs increase. The AN GWAS is still fairly immature, and in addition, our polygenic score had less power as we had to exclude the Swedish participants (*n* = 4,118 of 16,992 cases) from the discovery sample. Nevertheless, it had predictive capacity in our sample, especially in those individuals who were firmly anchored in an AN diagnosis only. The effect appears to be diluted in those individuals who display a more mixed eating disorders diagnostic picture across their illness journey. This may limit potential clinical utility in this subgroup of patients. The observed difference between those with AN only and those with a more mixed diagnostic picture also provides insight into how genetic factors may influence clinical presentation and course within the eating disorder diagnostic groups. These results are consistent with prior observations suggesting that the three primary eating disorders (AN, BN, and BED) differ on a genomic level [55]. AN PGS at initial presentation for treatment may hold promise in predicting who is likely to maintain an AN presentation versus experience diagnostic crossover, informing the tailoring of treatment accordingly. This should be investigated in larger independent samples as our results were inconsitent.

In our study, genetic liability to SCZ was not associated with disorder severity or long-term outcome in patients with AN. However, in the subsample of AN restricting patients only, the SCZ PGS showed a nominally significant (i.e., *p* < 0.05) negative association with the CIA score. This association did not remain significant after multiple testing correction, and was not detected at the main analysis *p* value threshold to generate the PGS of 0.001. The associations overall were inconsistent. Potentially, a larger target sample size providing greater statistical power may clarify these findings. The reason for a potential association between a higher SCZ polygenic load with a lower CIA score (i.e., less self-reported severity) is unclear, but genetic liability to SCZ could possibly be associated with poor illness insight, sometimes termed unintentional denial of illness or anosognosia, which is common in AN and often evidenced by unexpectedly low self-report of symptoms and distress relative to objective illness status [56, 57]. Future research could investigate further whether AN patients with high SCZ PGS represent a distinct phenotype with different treatment needs and response.

In our study, patients with AN carrying a greater BMI PGS experienced greater severity compared to eating disorder patients with low BMI PGSs. This finding stands in contrast to negative genetic correlation between BMI and AN as a diagnosis based on GWAS data [27, 55, 58]. However, the positive association between the BMI PGS and severity is consistent with findings that associated genetic liability to high BMI with engaging in more weight loss behaviours [59] and higher levels of disordered eating [60] in the general population. These studies [59, 60] and our finding support a shared genetic aetiology between genetic propensity for high BMI and eating disorder severity or specific symptoms. As fear of weight gain, a central symptom among individuals with some eating disorders, combined with genetic liability for high BMI might induce psychological distress, reflected as higher scores on the CIA in our study, future studies should investigate more refined measures of severity on the symptom level. Additionaly, our finding needs to be replicated in an independent sample.

## Limitations

Our findings must be interpreted in the light of several limitations. First, eating disorder diagnoses are entered at first registration to the national quality registers, meaning that some diagnostic crossover could have occurred prior to or after registration that was not captured in our data. Mitigating this limitation was the addition of the questionnaire-based lifetime data collected in the ED100K as part of the ANGI study that captures lifetime diagnostic migration. Second, we only included individuals with eating disorders who sought treatment. Although this increases clinical applicability, this might also have resulted in a more severe sample with less variance. Balancing that limitation, we included data from the majority of specialised eating disorder units across Sweden, capturing almost all Swedish individuals diagnosed with an eating disorder receiving all levels of care. Third, our measure of disorder severity is limited as we only used one continuous score at one time point. Future studies should include longitudinal assessments of severity and potentially pool different outcome variables to create a composite measure of severity. Fourth, participants were primarily of European (and Swedish) ancestry, which limits the generalisability to non-European populations. Fifth, the low number of males included in this study hinders our ability to address sex differences. Sixth, as the AN PGS was derived from a comparatively small GWAS excluding the Swedish participants, our statistical power to detect associations was limited. This is refelected in the inconsistent effect estimates across different *p* value thresholds at which the AN PGS was created (Fig. 1A-C) and additionally the inconsistent results when using the PRS-CS method to calculate PGSs. This phenomenon, however, had also been observed in studies of SCZ [61]. As AN most likely is polygenic and may have diverse underlying causes, one may hypothesise that more strongly associated genetic variants (i.e., lower generation *p* value thresholds) act via different biological pathways than less associated ones [62]. However, findings are mixed [61, 62]. It is anticipated that increasing the sample size of eating disorders GWASs will lead to the identification of more AN-associated genetic variants, as seen in other psychiatric disorders such as SCZ [63], boosting the statistical power, robustness, and clinical utility of the AN PGS [27]. Analyses like ours should be repeated when PGS with greater statistical power are available. Furthermore, different genetic variants may be implicated in the development of than in the maintenance of AN. Therefore, future GWASs should not only focus on a binary disorder phenotype, they should also consider the course of the disorder along the lifespan.

## Conclusion

No study of long-term outcome of eating disorders has included the role of genomic risk burden. Our study is the first to show an association between an AN and BMI PGS with severity or eating disorder outcome. We provide the first tentative evidence of potential clinical utility of PGSs [64] in the field of eating disorders. Our findings suggest that PGSs in combination with environmental variables may contribute to risk prediction models in AN, if GWAS sample sizes continue to increase. Ongoing concerns with slow progress in improving treatment outcomes, especially for AN [65, 66], call for advances in prevention, detection, and treatment. These results encourage the expansion of genetic studies of eating disorders to accelerate discovery and impact.

## Supplementary information

Supplementary Methods & Figures

Supplementary Tables

## Data Availability

Code to generate results is available upon request from the authors and made available on GitHub with publication.

## References

[1] Yilmaz Z, Hardaway JA, Bulik CM (2015). Genetics and epigenetics of eating disorders. Adv Genomics Genet.

[2] Arcelus J, Mitchell AJ, Wales J, Nielsen S (2011). Mortality rates in patients with anorexia nervosa and other eating disorders. A meta-analysis of 36 studies. Arch Gen Psychiatry.

[3] Fichter MM, Quadflieg N, Crosby RD, Koch S (2017). Long‐term outcome of anorexia nervosa: Results from a large clinical longitudinal study. Int J Eat Disord..

[4] Steinhausen H-C (2009). Outcome of eating disorders. Child Adolesc Psychiatr Clin N. Am.

[5] Noordenbos G, Oldenhave A, Muschter J, Terpstra N (2002). Characteristics and treatment of patients with chronic eating disorders. Eat Disord..

[6] Long CG, Fitzgerald K-A, Hollin CR (2012). Treatment of chronic anorexia nervosa: a 4-year follow-up of adult patients treated in an acute inpatient setting. Clin Psychol Psychother..

[7] Robinson PH. Severe and enduring eating disorder. John Wiley & Sons, Ltd; 2009.

[8] Andries A, Frystyk J, Flyvbjerg A, Støving RK (2014). Dronabinol in severe, enduring anorexia nervosa: a randomized controlled trial. Int J Eat Disord..

[9] Fox JRE, Diab P (2013). An exploration of the perceptions and experiences of living with chronic anorexia nervosa while an inpatient on an Eating Disorders Unit: An Interpretative Phenomenological Analysis (IPA) study. J Health Psychol..

[10] Dawson L, Rhodes P, Touyz S (2014). “Doing the impossible”: the process of recovery from chronic anorexia nervosa. Qual Health Res..

[11] Touyz S, Le Grange D, Lacey H, Hay P, Smith R, Maguire S (2013). Treating severe and enduring anorexia nervosa: a randomized controlled trial. Psychol Med..

[12] Arkell J, Robinson P (2008). A pilot case series using qualitative and quantitative methods: biological, psychological and social outcome in severe and enduring eating disorder (anorexia nervosa). Int J Eat Disord..

[13] Keski-Rahkonen A, Raevuori A, Bulik CM, Hoek HW, Rissanen A, Kaprio J (2014). Factors associated with recovery from anorexia nervosa: a population-based study. Int J Eat Disord..

[14] Zipfel S, Lowe B, Reas DL, Deter HC, Herzog W (2000). Long-term prognosis in anorexia nervosa: lessons from a 21-year follow-up study. Lancet (Lond, Engl).

[15] Löwe B, Zipfel S, Buchholz C, Dupont Y, Reas DL, Herzog W (2001). Long-term outcome of anorexia nervosa in a prospective 21-year follow-up study. Psychol Med..

[16] Lock J, Agras WS, Le Grange D, Couturier J, Safer D, Bryson SW (2013). Do end of treatment assessments predict outcome at follow-up in eating disorders?. Int J Eat Disord..

[17] Keel PK, Mitchell JE, Miller KB, Davis TL, Crow SJ (1999). Long-term outcome of Bulimia Nervosa. Arch Gen Psychiatry.

[18] Keel PK, Brown TA (2010). Update on course and outcome in eating disorders. Int J Eat Disord..

[19] Bulik CM, Sullivan PF, Joyce PR, Carter FA, McIntosh VV (1998). Predictors of 1-year treatment outcome in bulimia nervosa. Compr Psychiatry.

[20] Robinson P (2014). Severe and enduring eating disorders: recognition and management. Adv Psychiatr Treat.

[21] Berkman ND, Lohr KN, Bulik CM (2007). Outcomes of eating disorders: a systematic review of the literature. Int J Eat Disord..

[22] Steinhausen H-C (2002). The outcome of anorexia nervosa in the 20th century. Am J Psychiatry.

[23] Zhang R, Larsen JT, Kuja-Halkola R, Thornton L, Yao S, Larsson H, et al. Familial co-aggregation of schizophrenia and eating disorders in Sweden and Denmark. Mol. Psychiatry 2020. 10.1038/s41380-020-0749-x. 10.1038/s41380-020-0749-x 32382133

[24] Foulon C (2003). Schizophrenia and eating disorders. Encephale.

[25] Khalil RB, Hachem D, Richa S (2011). Eating disorders and schizophrenia in male patients: a review. Eat Weight Disord—Stud Anorex, Bulim Obes.

[26] Götestam KG, Eriksen L, Hagen H (1995). An epidemiological study of eating disorders in Norwegian psychiatric institutions. Int J Eat Disord..

[27] Watson HJ, Yilmaz Z, Thornton LM, Hübel C, Coleman JRI, Gaspar HA (2019). Genome-wide association study identifies eight risk loci and implicates metabo-psychiatric origins for anorexia nervosa. Nat Genet..

[28] Maier RM, Visscher PM, Robinson MR, Wray NR (2018). Embracing polygenicity: A review of methods and tools for psychiatric genetics research. Psychol Med..

[29] Howard DM, Adams MJ, Clarke T-K, Hafferty JD, Gibson J, Shirali M (2019). Genome-wide meta-analysis of depression identifies 102 independent variants and highlights the importance of the prefrontal brain regions. Nat Neurosci..

[30] Stahl EA, Breen G, Forstner AJ, McQuillin A, Ripke S, Trubetskoy V (2019). Genome-wide association study identifies 30 loci associated with bipolar disorder. Nat Genet..

[31] Ripke S, Neale BM, Corvin A, Walters JTR, Farh K-H, Holmans PA (2014). Biological insights from 108 schizophrenia-associated genetic loci. Nature.

[32] Bulik-Sullivan B, Finucane HK, Anttila V, Gusev A, Day FR, Loh P-R (2015). An atlas of genetic correlations across human diseases and traits. Nat Genet..

[33] Smoller JW, Andreassen OA, Edenberg HJ, Faraone SV, Glatt SJ, Kendler KS (2019). Psychiatric genetics and the structure of psychopathology. Mol Psychiatry.

[34] Bauer AE, Liu X, Byrne EM, Sullivan PF, Wray NR, Agerbo E (2019). Genetic risk scores for major psychiatric disorders and the risk of postpartum psychiatric disorders. Transl Psychiatry.

[35] Bohn K, Doll HA, Cooper Z, O’Connor M, Palmer RL, Fairburn CG (2008). The measurement of impairment due to eating disorder psychopathology. Behav Res Ther..

[36] Mayer LES, Roberto CA, Glasofer DR, Etu SF, Gallagher D, Wang J (2007). Does percent body fat predict outcome in anorexia nervosa?. Am J Psychiatry.

[37] Bodell LP, Mayer LES (2011). Percent body fat is a risk factor for relapse in anorexia nervosa: a replication study. Int J Eat Disord..

[38] El Ghoch M, Calugi S, Chignola E, Bazzani PV, Dalle Grave R (2016). Body mass index, body fat and risk factor of relapse in anorexia nervosa. Eur J Clin Nutr..

[39] Thornton LM, Munn-Chernoff MA, Baker JH, Jureus A, Parker R, Henders AK (2018). The Anorexia Nervosa Genetics Initiative (ANGI): overview and methods. Contemp Clin Trials.

[40] Birgegard A, Bjorck C, Clinton D (2010). Quality assurance of specialised treatment of eating disorders using large-scale Internet-based collection systems: methods, results and lessons learned from designing the Stepwise database. Eur Eat Disord Rev..

[41] Schaumberg K, Jangmo A, Thornton LM, Birgegård A, Almqvist C, Norring C (2019). Patterns of diagnostic transition in eating disorders: a longitudinal population study in Sweden. Psychol Med.

[42] Birgegård A, De Man Lapidoth J. Validation of the structured eating disorder interview (SEDI) against the eating disorder examination (EDE). Sockholm: Karolinska Institutet; 2010.

[43] First MB, Gibbon M, Spitzer RL, Williams JBW. Structured clinical interview for DSM-IV Axis I disorders (J. Herlofson Trans.)*.* Danderyd: Pilgrim Press; 1998.

[44] Kearney CA, Freeman A, Bacon V. 11—Structured and semistructured interviews for children. In: Goldstein G, Allen DN, DeLuca J. editors. Handbook of psychological assessment, 4th ed. Academic Press, 337–53; 2019. 10.1016/B978-0-12-802203-0.00011-0.

[45] Holgado–Tello FP, Chacón–Moscoso S, Barbero–García I, Vila–Abad E (2008). Polychoric versus Pearson correlations in exploratory and confirmatory factor analysis of ordinal variables. Qual Quant..

[46] Ekeroth K, Birgegård A (2014). Evaluating reliable and clinically significant change in eating disorders: comparisons to changes in DSM-IV diagnoses. Psychiatry Res..

[47] Yengo L, Sidorenko J, Kemper KE, Zheng Z, Wood AR, Weedon MN (2018). Meta-analysis of genome-wide association studies for height and body mass index in ∼700000 individuals of European ancestry. Hum Mol Genet.

[48] Choi SW, O’Reilly PF. PRSice-2: Polygenic Risk Score software for biobank-scale data. Gigascience. 2019;8:giz082. 10.1093/gigascience/giz082 PMC6629542 31307061

[49] Price AL, Patterson NJ, Plenge RM, Weinblatt ME, Shadick NA, Reich D (2006). Principal components analysis corrects for stratification in genome-wide association studies. Nat Genet..

[50] Purcell S, Neale B, Todd-Brown K, Thomas L, Ferreira MAR, Bender D (2007). PLINK: a tool set for whole-genome association and population-based linkage analyses. Am J Hum Genet..

[51] Wray NR, Lee SH, Mehta D, Vinkhuyzen AAE, Dudbridge F, Middeldorp CM (2014). Research review: polygenic methods and their application to psychiatric traits. J Child Psychol Psychiatry..

[52] Murray GK, Lin T, Austin J, McGrath JJ, Hickie IB, Wray NR. Could polygenic risk scores be useful in psychiatry?: a review. JAMA Psychiatry. 2020. 10.1001/jamapsychiatry.2020.3042. 10.1001/jamapsychiatry.2020.3042 33052393

[53] Levine ME, Crimmins EM, Prescott CA, Phillips D, Arpawong TE, Lee J (2014). A polygenic risk score associated with measures of depressive symptoms among older adults. Biodemography Soc Biol..

[54] Zhang J-P, Robinson D, Yu J, Gallego J, Fleischhacker WW, Kahn RS (2019). Schizophrenia polygenic risk score as a predictor of antipsychotic efficacy in first-episode psychosis. Am J Psychiatry.

[55] Hübel C, Abdulkadir M, Herle M, Loos RJF, Breen G, Bulik CM, Micali N (2021). One size does not fit all. Genomics differentiates among anorexia nervosa, bulimia nervosa, and binge-eating disorder. Int J Eat Disord.

[56] Vandereycken W (2006). Denial of illness in anorexia nervosa—a conceptual review: part 1 diagnostic significance and assessment. Eur Eat Disord Rev..

[57] Vandereycken W (2006). Denial of illness in anorexia nervosa—a conceptual review: part 2 different forms and meanings. Eur Eat Disord Rev..

[58] Duncan L, Yilmaz Z, Gaspar H, Walters R, Goldstein J, Anttila V (2017). Significant locus and metabolic genetic correlations revealed in genome-wide association study of anorexia nervosa. Am J Psychiatry.

[59] Nagata JM, Braudt DB, Domingue BW, Bibbins-Domingo K, Garber AK, Griffiths S (2019). Genetic risk, body mass index, and weight control behaviors: Unlocking the triad. Int J Eat Disord.

[60] Abdulkadir M, Herle M, De Stavola BL, Hübel C, Santos Ferreira DL, Loos RJF (2020). Polygenic score for body mass index is associated with disordered eating in a general population cohort. J Clin Med..

[61] Ursini G, Punzi G, Chen Q, Marenco S, Robinson JF, Porcelli A (2018). Convergence of placenta biology and genetic risk for schizophrenia. Nat Med..

[62] Vassos E, Kou J, Tosato S, Maxwell J, Dennison CA, Legge SE, et al. Lack of Support for the Genes by Early Environment Interaction Hypothesis in the Pathogenesis of Schizophrenia. Schizophr Bull. 2021. 10.1093/schbul/sbab052. 10.1093/schbul/sbab052 PMC8781344 33987677

[63] Kim Y, Zerwas S, Trace SE, Sullivan PF (2011). Schizophrenia genetics: where next?. Schizophr Bull..

[64] Lewis CM, Vassos E (2017). Prospects for using risk scores in polygenic medicine. Genome Med.

[65] Wonderlich SA, Bulik CM, Schmidt U, Steiger H, Hoek HW (2020). Severe and enduring anorexia nervosa: Update and observations about the current clinical reality. Int J Eat Disord..

[66] Kaye WH, Bulik CM. A crisis in care: the treatment of anorexia nervosa in the US. JAMA Psychiatry. 2021;78:591–2. 10.1001/jamapsychiatry.2020.4796 33625500

