# Supplementary material for: Polygenic association with severity and long-term outcome in eating disorder cases
Source: Transl Psychiatry. 2022 Feb 16;12:61. doi: 10.1038/s41398-022-01831-2 (PMC8850420; doi:10.1038/s41398-022-01831-2)
Supplement: Supplementary file 1 — Supplementary Methods & Figures [file 41398_2022_1831_MOESM1_ESM.docx]

**Polygenic association with severity and long-term outcome in eating disorder cases**

**Therese Johansson, Andreas Birgegård, Ruyue Zhang, Sarah Bergen, Mikael Landén, Liselotte Vogdrup Petersen, Cynthia M. Bulik, Christopher Hübel**

**Supplementary Material**

**Supplementary Methods:**

**Clinical Impairment Assessment Questionnaire (CIA) description**

**Supplementary Figures:**

**Supplementary Figure S1A-D.** Sixteen items of the Clinical Impairment Assessment (CIA) presented as a Likert plot and a boxplot (**S1D**) presenting median CIA total score among the different groups analysed

**Supplementary Figure S2.** Correlation matrix displaying correlation coefficients between clinical impairment assessment (CIA) items 1 to 16 and CIA global, personal, social, and cognitive score

**Supplementary Figure S3A-C.** Correlation matrices between polygenic scores:

A anorexia nervosa, B schizophrenia, C body mass index

**Supplementary Figure S4.** Correlation matrix displaying correlation coefficients between independent and dependent variables used in regression analyses

**Supplementary Methods**

**Clinical Impairment Assessment (CIA) Questionnaire.** The CIA questionnaires test-retest reliability (ICC = 0.86, 95% CI: 0.75; 0.92; *p* < 0.001), construct validity (rs = 0.89, *p* < 0.001) internal consistency (Cronbach’s alpha = 0.97), sensitivity to change and ability to predict eating disorder case status have been evaluated in the context of a treatment trial and was reported to be satisfactory. A receiver Operating Curve analysis showed that global CIA score was better able to predict case status (area under the curve = 0.88) than the three individual domains (personal, social, and cognitive domains 0.87, 0.85, and 0.80.^1^ Ekeroth & Birgegård (2014)^2^ proposed that the best cut-off point to predict case status was a global CIA score of 18 for patients with anorexia nervosa. In the present sample, internal consistency reliability was assessed using McDonald’s omega coefficients.^3^ The reliability estimates for the overall variance and for the variance owing to the general factor in the present sample data were ωt = 0.98 and ωh = 0.91, respectively.

**Supplementary Figure S2A-C. Sixteen items of the Clinical Impairment Assessment (CIA) presented as a Likert plot.**

**Figure S1A** is based on the overall sample including 2,873 individuals with anorexia nervosa that also experienced additional eating disorders presentations during their course of illness. All individuals were registered to receive specialised eating disorders care in Sweden. The Figure shows percentages of self-report responses to the 16 items rated on a 4-point Likert scale. The first six items cover personal impairment, the next five social impairment, and the last five cognitive impairment.

**Figure S1B** is based on the 1,839 individuals with clinically ascertained anorexia nervosa diagnosis. This is a subsample of the original sample with 2,873 individuals registered to receive specialised eating disorder care in Sweden. The Figure shows percentages of self-report responses to the 16 items rated on a 4-point Likert scale. The first six items cover personal impairment, the next five social impairment, and the last five cognitive impairment.

**Figure S1C** is based on the 976 individuals with clinically ascertained anorexia nervosa restricting subtype diagnosis a subsample of individuals registered to receive specialised eating disorder care in Sweden. The Figure shows percentages of self-report responses to the 16 items rated on a 4-point Likert scale. The first six items cover personal impairment, the next five social impairment, and the last five cognitive impairment.


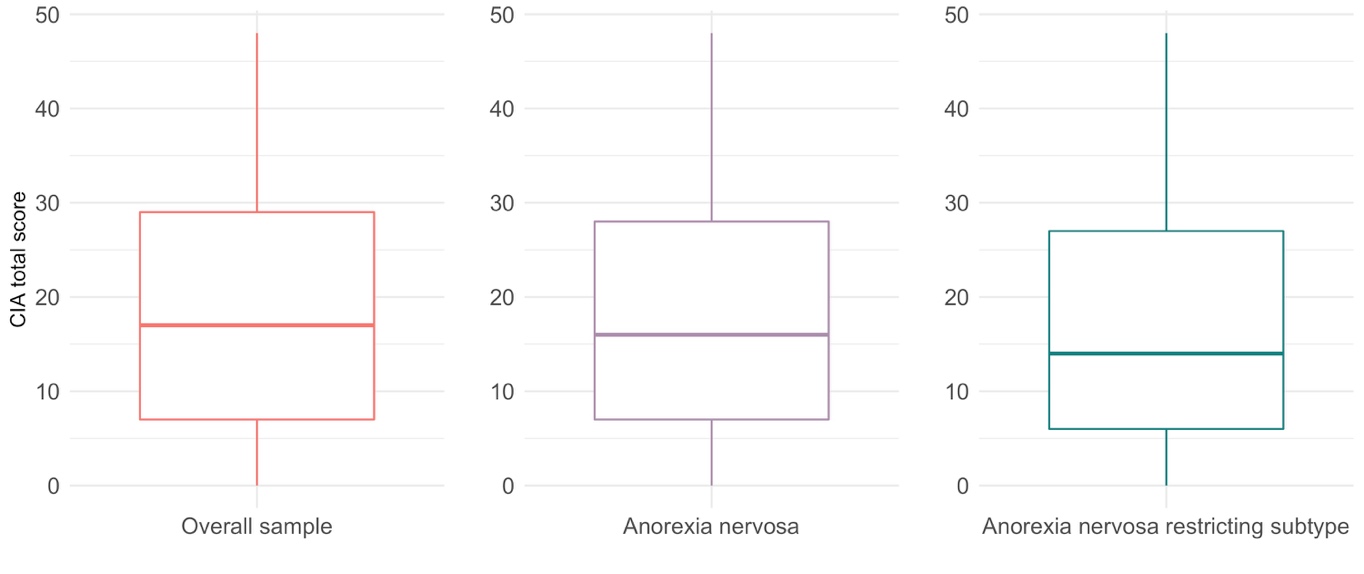


**Figure S1D** shows box plots of median clinical impairment assessment (CIA) total score by each sample analysed; the overall sample based on those 2,873 individuals with anorexia nervosa that also experienced additional eating disorders presentations during their course of illness, the 1,839 individuals with clinically ascertained anorexia nervosa diagnosis, and the 976 individuals with clinically ascertained anorexia nervosa restricting subtype diagnosis. Within each box, horizontal lines denote median values; boxes extend from 25th to the 75th percentile of each group's distribution of values.

**Supplementary Figure S2. Correlation matrix displaying correlation coefficients between clinical impairment assessment (CIA) items 1 to 16 and CIA global, personal, social, and cognitive score**Item 2, 8, 9, 11, 14 and 16 cover personal impairment. Item 3, 7, 10, 12 and 15 cover social impairment. Item 1, 4, 5, 6 and 13 cover cognitive impairment. Correlation between each item (1-16) were assessed using polychoric correlations. Correlations between items and sum scores (global, personal, social, and cognitive) were assessed using polyserial correlations, and correlation between sum scores were assessed using Pearson’s correlations.

**
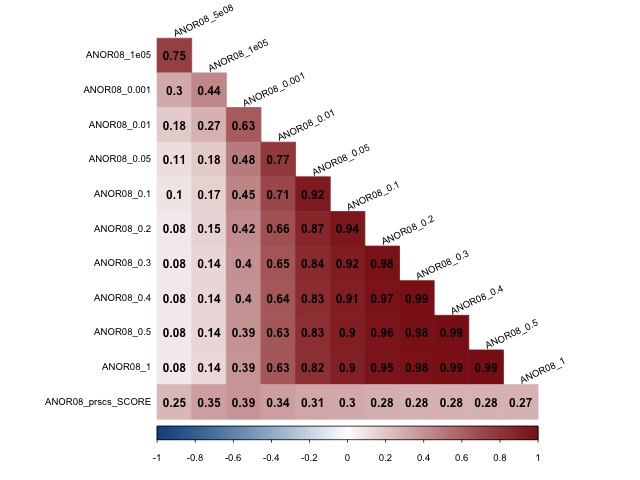
**

**Supplementary Figure S3A.** Correlation matrix between polygenic scores (PGS) for anorexia nervosa (AN) either calculated using different p value thresholds of the discovery genome-wide association study (GWAS) or the polygenic risk score continuous shrinkage (PRS-CS) method.

ANOR08 = internal code for AN GWAS without the Swedish sample

prscs = polygenic risk score continuous shrinkage

**
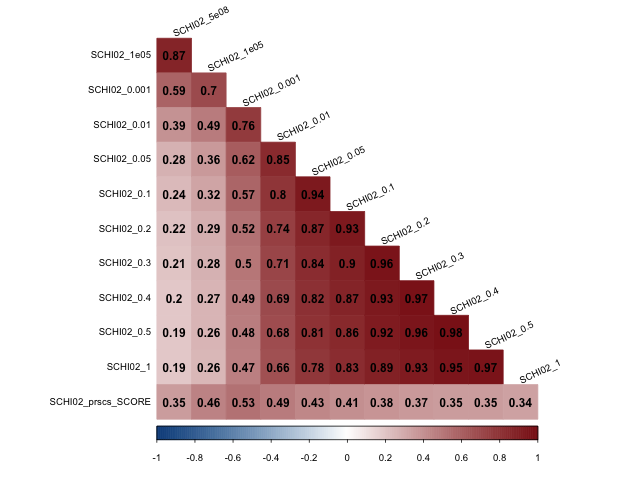
**

**Supplementary Figure S3B.** Correlation matrix between polygenic scores (PGS) for schizophrenia (SCZ) either calculated using different p value thresholds of the discovery genome-wide association study (GWAS) or the polygenic risk score continuous shrinkage (PRS-CS) method.

SCHI02 = internal code for schizophrenia GWAS without the Swedish sample

prscs = polygenic risk score continuous shrinkage

**
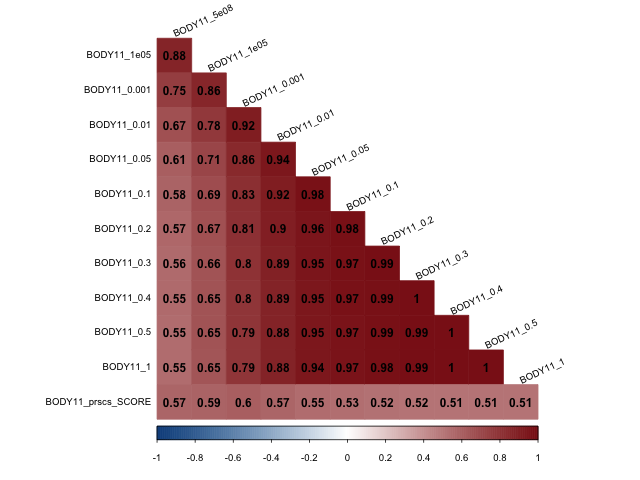
**

**Supplementary Figure S3C.** Correlation matrix between polygenic scores (PGS) for body mass index (BMI) either calculated using different p value thresholds of the discovery genome-wide association study (GWAS) or the polygenic risk score continuous shrinkage (PRS-CS) method.

BODY11 = internal code for body mass index GWAS without the Swedish sample

prscs = polygenic risk score continuous shrinkage

**Supplementary Figure S4. Correlation matrix displaying correlation coefficients between independent and dependent variables used in regression analysis.**

Correlation between numerical variables were assessed using Pearson’s correlations, correlation between categorical variables were assessed using polychoric correlations, and polyserial correlations were used to assess the correlation between age, age at eating disorder (ED) onset, follow-up time (i.e., numeric variables), and treatment unit, sex, and eating disorder diagnosis (i.e., categorical variables).

Nonsignificant correlations (α > 0.05) are shown in white
*CIA* Clinical Impairment Assessment
*BMI* Body mass index, constructed from height and weight measurements at first registration

**References**

1. Bohn, K. *et al.* The measurement of impairment due to eating disorder psychopathology. *Behav. Res. Ther.* **46**, 1105–1110 (2008).

2. Ekeroth, K. & Birgegård, A. Author ’ s personal copy Evaluating reliable and clinically signi fi cant change in eating disorders : Comparisons to changes in DSM-IV diagnoses.

3. Dunn, T. J., Baguley, T. & Brunsden, V. From alpha to omega: A practical solution to the pervasive problem of internal consistency estimation. *Br. J. Psychol.* **105**, 399–412 (2014).
